# Quantification of Drugs in Distinctly Separated Ocular Substructures of Albino and Pigmented Rats

**DOI:** 10.3390/pharmaceutics12121174

**Published:** 2020-12-02

**Authors:** Anna-Kaisa Rimpelä, Michel Garneau, Katja S. Baum-Kroker, Tanja Schönberger, Frank Runge, Achim Sauer

**Affiliations:** Department of Drug Discovery Sciences, Boehringer Ingelheim Pharma GmbH & Co. KG, 88397 Biberach, Germany; anna-kaisa.rimpelae@boehringer-ingelheim.com (A.-K.R.); michel.garneau@boehringer-ingelheim.com (M.G.); katja.baum-kroker@boehringer-ingelheim.com (K.S.B.-K.); tanja.schoenberger@boehringer-ingelheim.com (T.S.); frank.runge@boehringer-ingelheim.com (F.R.)

**Keywords:** ocular, drug delivery, pharmacokinetics, tissue isolation, rat, eye, drug concentration, method, pigment, melanin

## Abstract

The rat is a commonly used species in ocular drug research. Detailed methods of separating rat ocular tissues have not been described in literature. To understand the intraocular drug distribution, we developed a robust method for the separation of individual anterior and posterior substructures of pigmented Brown Norway (BN) and albino Wistar Han (WH) rat eyes, followed by quantification of drug concentration in these substructures. A short formalin incubation, which did not interfere with drug quantification, enabled the preservation of individual tissue sections while minimizing cross-tissue contamination, as demonstrated by histological analysis. Following oral administration, we applied the tissue separation method, in order to determine the ocular concentrations of dexamethasone and levofloxacin, as well as two in-house molecules BI 113823 and BI 1026706, compounds differing in their melanin binding. The inter-individual variability in tissue partitioning coefficients (Kp) was low, demonstrating the reproducibility of the separation method. Kp values of individual tissues varied up to 100-fold in WH and up to 46,000-fold in BN rats highlighting the importance of measuring concentration directly from the ocular tissue of interest. Additionally, clear differences were observed in the BN rat tissue partitioning compared to the WH rat. Overall, the developed method enables a reliable determination of small molecule drug concentrations in ocular tissues to support ocular drug research and development.

## 1. Introduction

Diseases of the posterior segment of the eye are the leading causes of blindness in many western countries [1,2]. Treating these diseases, however, remains challenging, due to limitations in available drugs and drug delivery methods. For many posterior segment diseases, such as the dry form of age-related macular degeneration (AMD) and normotensive glaucoma, there are no treatments available. For others, such as the wet form of AMD, diabetic retinopathy and macular edema, treatments are delivered through frequent intravitreal injections. So far, no clinical successes have been seen in developing oral or other less invasive treatments for these diseases. Therefore, there is a tremendous need to develop new therapies and improve drug delivery methods for posterior segment diseases. These challenges are being addressed with a wide variety of academic and industrial research efforts in exploring new delivery routes and developing improved formulations to deliver the drug to the posterior segment less invasively [3,4,5]. In addition, new therapeutic concepts are being evaluated in various animal models [6,7] and attempts are being made to repurpose old active molecules for ocular indications [8].

Ocular drug research requires information on local ocular drug concentrations, whether it is to assess drug release from formulations, in order to evaluate pharmacokinetics of ocular drugs or to assess their efficacy in animal models. An important pre-requisite for developing a successful new treatment is to deliver the drug to the target tissue at therapeutic concentrations. The eye is a pharmacokinetically complex organ, with a multitude of tissue barriers and uneven drug distribution into its substructures. For small molecule drugs, accumulation caused by melanin binding in the highly pigmented retinal pigment epithelium (RPE) and choroid in the posterior segment, as well as the iris and ciliary body (CB) in the anterior segment of the eye can result in large concentration differences between pigmented and non-pigmented tissues [9,10]. As targets for posterior segment diseases are located in both, the pigmented and non-pigmented tissues (neural retina, RPE and choroid), it is crucial to evaluate the concentration of the drug in the specific target tissue, enabling a thorough assessment and refinement of pharmacokinetic/pharmacodynamic (PK/PD) relationships of ocular drug candidates.

Despite its small eye size, the rat is a commonly used model species in ocular drug research [11]. Due to wide availability, easy handling, relatively low cost and ethical reasons, various disease models have been developed in the rat. For the same reasons, the rat is also used for early ocular pharmacokinetic evaluations. To date, only limited literature is available on the methods of tissue separation of the rat eye. Previous studies on ocular concentrations in the rat, either consider the eyes as a whole [12], differentiate only between a few ocular tissues [13,14,15] or refrain from describing tissue separation methods in detail [9]. The rabbit is another frequently used species in ocular studies, but the information on the methodology used for tissue separation of the rabbit eye is also limited [16]. Due to the small size of the rat eye, the separation process is more delicate compared to the rabbit eye and the risk of cross-tissue contamination is higher. For melanin-binding drugs, a contamination originating from the pigmented tissues, can lead to considerable overestimation of drug concentration in non-pigmented tissues. To the best of our knowledge, this contamination has not been considered in the literature in any animal species previously. For the various reasons listed above, there is a significant need for a reliable and reproducible method to separate the distinct substructures of the rat eye, overcoming the limitations and lack of method description of previously reported studies.

The aim of the present study was to develop a method for the separation of rat ocular tissues and their processing for quantification of small molecule drug concentrations. As both albino and pigmented rats are used in pre-clinical research, understanding the impact of melanin binding on ocular distribution is highly important for small molecule drugs. Consequently, we developed the method in albino Wistar Han (WH) and pigmented Brown Norway (BN) rats and demonstrated its functionality in vivo with four compounds. The method enables the quantification of drug concentrations from individual ocular tissues of the rat eye while the obtained results emphasize the importance of measuring tissue-specific concentrations.

## 2. Materials and Methods

### 2.1. Chemicals

Dexamethasone (purity ≥ 97%) and levofloxacin (purity ≥ 98%) were obtained from Sigma Aldrich (St. Louis, MO, USA) and BI 113823, as well as BI 1026706 from the in-house dispensary at Boehringer Ingelheim (BI). Melanin from *Sepia officinalis* and synthetic melanin were obtained from Sigma Aldrich together with sodium hydroxide (NaOH), bovine serum albumin (BSA) and formalin solution (10%, neutral buffered). Dimethyl sulfoxide (DMSO) was purchased from Merck Millipore (Burlington, MA, USA). Phosphate buffered saline (PBS) was made from one vial of phosphate buffer powder (pH 6.6, Sigma Aldrich) and 34.2 g of NaCl (Sigma Aldrich) by mixing both in purified water (Milli-Q, MilliporeSigma, Burlington, MA, USA) in a total volume of 3.8 L. The pH was adjusted to 6.5 with HCl. Ethanol (EtOH) was obtained from Carl Roth GmbH (Karlsruhe, Germany). Acetonitrile (ACN) was purchased from Honeywell (Charlotte, NC, USA). Tween-80 (Polysorbat 80) and hydroxyethyl cellulose (Natrosol) for the oral suspension formulation of the drugs were obtained from Dr. W. Kolb AG (Hedingen, Switzerland) and Ashland Industries Deutschland (Düsseldorf, Germany), respectively.

### 2.2. Animals

All animal experiments were conducted in accordance with the German and European Animal Welfare Act and authorized by the Regierungspräsidium Tübingen as the responsible local German authority under reference numbers 14-009-G (25 June 2014) and 19-004-G (04 June 2019). Animal experiments were performed in male Wistar Han (Crl:WI(Han)) and Brown Norway (BN/Crl) rats (Charles River Research Models and Services Germany GmbH). The average weight of the animals was 220 g (range 204–260 g). The animals were housed under a 12-h light/dark cycle with free access to standardized pelleted food (Granovit AG) and water.

### 2.3. Ocular Tissue Distribution Study

Cocktails of either dexamethasone (4 mg/kg) and levofloxacin (7 mg/kg) (cocktail 1) or the two internal Boehringer Ingelheim compounds BI 113823 (10 mg/kg) and BI 1026706 (10 mg/kg) (cocktail 2) were administered to the rats via oral gavage (n = 3 rats/strain for cocktail 1 and n = 2 rats/strain for cocktail 2) at a dose volume of 2 mL/kg. The compounds were dosed as a suspension in a vehicle consisting of 0.015% Tween-80 and 0.5% hydroxyethyl cellulose in water. Blood samples were obtained under isoflurane anesthesia at 0.25, 0.5, 1, 2, 4, and 8 h. Blood was collected from the sublingual vein into K_3_EDTA coated vials (Sarstedt, Nümbrecht, Germany) and plasma was obtained by centrifugation for 5 min at approximately 3500× *g*. At 8 h, the anesthetized animals were euthanized by anesthetic overdose followed by exsanguination, and ocular tissues were collected, as described below. In addition, a small piece of the femoral muscle was excised as a reference tissue for tissue distribution as previously described by Cui et al. [17].

### 2.4. Ocular Tissue Separation and Sampling

Ocular tissue separation was performed under microscope control (Carl Zeiss f-170 Stereoscope, Oberkochen, Germany). Immediately following euthanasia, the aqueous and vitreous humor were collected. The aqueous humor was sampled via an insulin syringe (30 G) introduced through the cornea into the anterior chamber, transferred to a vial and frozen pending analysis. A sample from the vitreous humor was collected via a glass capillary using a modification of the mousetap technique [18]. A 20 G needle was used to puncture the posterior segment of the eye (2 mm posterior to the limbus). A glass microelectrode with a broken tip (borosilicate glass, wall thickness 0.225 mm, outside diameter 1.5 mm; Hilgenberg GmbH, Malsfeld, Germany; pulled with a DMZ-Universal Puller, Zeitz-Instruments GmbH, Munich, Germany) was inserted posterior to the lens. Approximately 6 µL of vitreous humor was slowly aspirated into the glass microelectrode via silicone tubing, connected to a low volume syringe, placed in a vial and frozen.

**Scheme 1:**
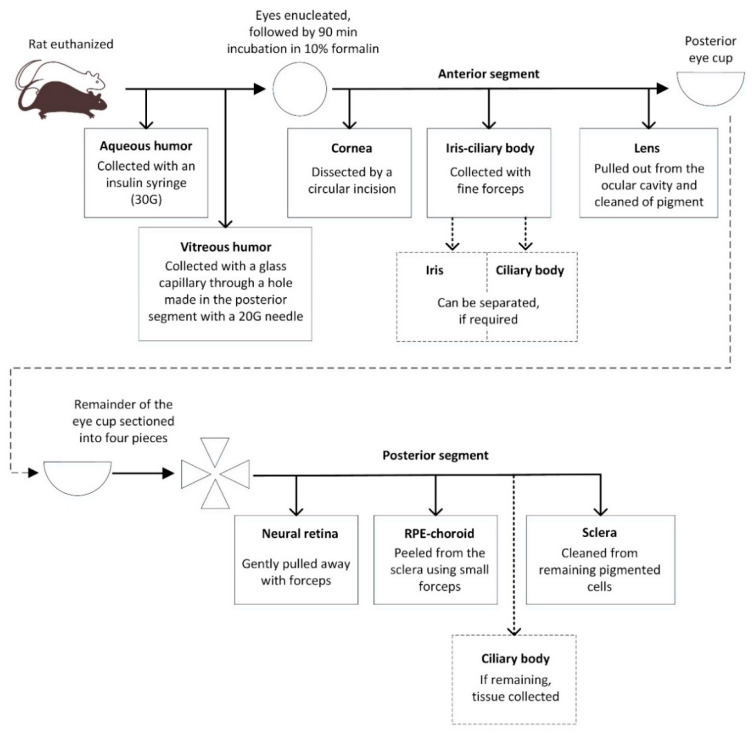
Ocular tissue separation procedure. RPE = retinal pigmented epithelium.

The eye was then carefully enucleated, any extraocular material was removed, and the eye was fixed in 2 mL of 10% formalin for 90 min at room temperature. After fixation, the eye was blotted dry to remove excess formalin and placed on a holding block with the cornea facing up. Under the dissection microscope, the cornea was dissected by a circular incision in the limbus region. It was then removed and cleaned of other tissues. The iris and pieces of the ciliary body were separated from other tissues (cornea and/or lens) using a pair of fine forceps. The lens was pulled out of the ocular cavity and freed of any remaining contaminating tissue. The remainder of the eye cup was placed on a flat surface. Starting from the optic nerve toward the ora serrata, the eye was carefully sectioned into four pieces using a scalpel. If any vitreous humor was still present, it was carefully removed. Using a pair of fine forceps, the neural retina was detached from the eye at the optical nerve junction and at the ora serrata. The neural retina was gently pulled away from the eye. Heavily contaminated retinal sections were discarded and any residual pigmented tissues removed with fine forceps. The RPE-choroid was peeled from the sclera using small forceps. Care was taken not to collect the remaining part of the ciliary body that might have been left attached to the eye; the remaining ciliary body was collected and added to the iris-CB sample. The remaining scleral tissue was cleaned from adherent pigmented cells by wiping with a cotton swap. All the dissected tissues were placed in pre-weighed tubes. All tubes were weighed and the tissue weights recorded. The step-by-step tissue separation process is described in Scheme 1 and illustrated in Figure S1.

### 2.5. Effect of Formalin Fixation on Drug Recovery

We tested the effect of formalin fixation on drug recovery by performing an additional ocular distribution study with cocktail 1 compounds (same study design as described above) in both rat strains (n = 3 rats/strain). At the 8 h timepoint, aqueous and vitreous humor collection followed by enucleation was performed as described above. The right eye was immediately separated in the distinct ocular tissues (cornea, iris-CB and lens). The posterior part of the eye was kept intact (retina, RPE-choroid and sclera), as a clean separation of the tissues from the fresh eye was not achievable. All tissues were weighed and frozen until analysis. The entire left eye was immediately added to 2 mL of 10% formalin. Following the 90-min formalin incubation, the cornea, iris-CB and lens of the left eye were isolated as described above while the posterior section (retina, RPE-choroid and sclera) was kept as a whole. All tissues were weighed and frozen pending analysis.

### 2.6. Melanin Binding and Drug Extraction from Melanin

We measured the melanin binding of the compounds in vitro with melanin from *Sepia officinalis* applying a previously described method [19]. The compounds (1 µM) were incubated with Sepia melanin (1 mg/mL in PBS pH 6.5) in a volume of 500 µL. After 2 h of incubation, the tubes were centrifuged at 20,000× *g* for 15 min to pellet the melanin with the bound drug, and the supernatant was sampled for quantification of the unbound drug concentration. We measured the extraction of the compounds from the melanin-drug pellets, first with a standard tissue extraction solvent (EtOH:H_2_O (4:1)), and thereafter, with a basic solvent (100 mM NH_4_OH in EtOH:H_2_O (4:1)) in case the extraction was low with the first solvent. The supernatant was fully removed and the pellet was resuspended in 500 µL of the solvent. The suspension was then moved to a 2-mL Precellys^®^ homogenization tube (CKMix, Bertin Instruments, Montigny-le-Bretonneux, France) and homogenized with a Precellys^®^ Evolution homogenizer (Bertin Instruments, Montigny-le-Bretonneux, France) at 5500 rpm (three cycles of 40 s with a 30 s break in between) to replicate the process to be used for the in vivo tissue samples. After homogenization, the tubes were centrifuged at 14,200× *g* for 10 min. A sample from the supernatant was taken for the analysis of the amount extracted from the melanin and subsequent calculation of the extraction rate. For cocktail 1 drugs, this process was repeated twice to assess if more compound could be extracted by repeated washing. Control samples for the analysis of the total drug concentration were incubated in the same manner as in the binding assay, but the melanin suspension was replaced with PBS (pH 6.5) containing 0.1% BSA to prevent nonspecific binding of the compounds to the tube.

The samples were analyzed by high performance liquid chromatography coupled to tandem mass spectrometry (HPLC-MS/MS) with a similar method described later for the tissue samples. The samples were prepared for the analysis by mixing 25 µL of the sample with 50 µL of ACN:H_2_O (4:1), 25 µL of an internal standard solution, 25 µL of blank matrix (either PBS with 0.1% BSA or PBS pre-incubated with melanin), and 200 µL of ACN. The prepared samples were then centrifuged at 3200× *g* for 10 min, and a 120 µL sample of the supernatant was mixed with 120 µL of ACN:H_2_O (4:1). The identified extraction solvents were later applied to the in vivo tissue samples.

### 2.7. Tissue Sample Preparation and Analysis

Pre-weighed ocular tissue samples were homogenized with a Precellys^®^ Evolution homogenizer in the same manner as for the compound extraction from melanin. The extraction solvent used for each cocktail was selected based on the in vitro extraction study described above: 100 mM NH_4_OH in EtOH:H_2_O (4:1) was used for cocktail 1 (dexamethasone, levofloxacin) and EtOH:H_2_O (4:1) for cocktail 2 (BI 113823, BI 1026706). Extraction volume (i.e., extraction ratio) depended on the tissue and ranged from 10 to 250 µL/mg tissue. Detailed information can be found in Supplementary Material (Table S1). After the homogenization, the samples were centrifuged at 14,200× *g*. Internal standard in ACN or ACN:methanol (1:1) was added to an aliquot of the supernatant before sample analysis. The plasma samples were directly diluted with the internal standard, centrifuged, and further diluted 1:2 with 0.1% formic acid. To avoid unnecessary use of animals, standard curves prepared in WH rat plasma with the same pre-treatment as used for the plasma samples and used for all plasma and tissue samples. To confirm the validity of the standard curve for specific tissue samples, quality control (QC) samples were prepared in appropriate strain-specific tissues at three different concentrations; the tissue was homogenized, as described above for the tissue samples and the homogenate was spiked with the compound.

Drug concentrations in the samples were determined by HPLC-MS/MS. BI 113823 and BI 1026706 were quantified using [^13^C_2_D_2_]BI 113823 and [^13^CD_2_^15^N]BI 1026706 as internal standards. Dexamethasone and levofloxacin were quantified without an internal standard. Calibration ranges for each compound are listed in Table S2. The assays comprised sample clean-up by protein precipitation, chromatography with gradient elution on a 1260 Infinity II HPLC-system (Agilent Technologies, Waldbronn, Germany) equipped with a Waters Acquity HSS T3, 2.1 × 50 mm, 1.8 µm analytical HPLC column (BI compounds) or a Phenomenex Luna Omega Polar C18, 2.1 × 50 mm, 1.6 µm column (dexamethasone, levofloxacin) and detection and quantification of the analytes by electrospray ionisation in the positive ion mode on a API6500 (AB (SCIEX, Concord, ON, Canada). For quantification, transitions from m/z = 525.3 → 181.2 and m/z = 529.3 → 181.3 were recorded for BI 113823 and its internal standard, respectively, m/z = 536.2 → 155.1 and m/z = 540.0 → 156.1 for BI 1026706 and its internal standard, respectively. The transitions m/z = 393.1 → 373.1 and m/z = 362.1 → 318.1 were recorded for dexamethasone, and levofloxacin, respectively. Concentration results are reported as nmol/L (nM) assuming a tissue density of 1 g/mL.

Tissue/plasma concentration ratios, i.e., tissue partition coefficients (*Kp_tissue_*), were calculated of the concentrations based on Equation (1), where *C_tissue_* refers to the concentration in the tissue and *C_plasma_* to plasma concentration. The ratio of tissue partitioning between *BN* and *WH* rat (*BN/WH ratio*) was calculated according to Equation (2):

(1) $$Kp_{tissue}=C_{tissue}/C_{plasma}$$

(2) $$BN/WHratio=Kp_{tissue,BN}/Kp_{tissue,WH}$$

### 2.8. Histology

To investigate the level of cross-tissue contamination, we prepared the ocular tissues for histological examination using the above-described protocol. After separation of the different ocular layers, the tissues were cut into smaller pieces and transferred into histology cassettes. To avoid sample loss and to keep the samples flat, sponges and filter paper were used. The samples were fixed in 10% neutral buffered formalin for approximately 24 h, processed with an automated tissue processor (Tissue-Tek^®^ VIP^®^ 6, Sakura Finetek Europe B.V., Alphen aan den Rifn, the Netherlands), embedded in paraffin and cut in 3 µm sections. Staining with hematoxylin and eosin (HE) was performed according to standard protocols.

### 2.9. Melanin Contamination in the Neural Retina

To estimate the possible contamination in the neural retina from the underlying, highly pigmented RPE-choroid in the BN rat, we measured the melanin content of pooled retinal samples (two replicates of two pooled retinas) where no measures were taken to avoid contamination in the isolation process (i.e., the retinal tissue sample was not cleaned of remaining pigmented tissue). Melanin content was determined using the sodium hydroxide solubilization method [20,21,22] with small modifications. The isolated retina was diluted 1:40 (*w:v*) with EtOH:H_2_O (4:1) and homogenized with Precellys^®^ Evolution homogenizer at 5500 rpm (three cycles of 40 s with a 30 s break in between). The homogenization cycle was run twice to assure a homogeneous melanin concentration. An aliquot of the homogenate was then mixed with 1.5 parts of 1.7 M NaOH:DMSO (9:1) and the samples were heated at 90–95 °C for 30 min to solubilize the tissue and melanin. The absorbance of the sample was immediately measured at 475 nm with SpectraMax 340PC384 microplate reader (Molecular Devices, San Jose, CA, USA) using a Nunc MaxiSorp 96-well plate (Thermo Fisher Scientific, Waltham, MA, USA) with an 80 µL sample volume.

We made a calibration curve for the retinal melanin contamination measurement by spiking albino (WH) rat retina with RPE-choroid from the pigmented BN rat. Both tissues were individually homogenized as mentioned above, and 1.5 parts of 1.7 M NaOH:DMSO (9:1) was added to aliquots of the homogenates. The albino retina was then spiked with pigmented RPE-choroid before heating these spiked calibration samples at 90–95 °C for 30 min and measuring the absorbance as described for the retinal samples above. The lower limits of quantification (*LLOQ*) and detection (*LLOD*) were determined from the absorbance of 14 blank samples (albino retina without added RPE-choroid) as follows:

(3) LLOQ=mean+10·SD

(4) LLOD=mean+3.3·SD

## 3. Results

### 3.1. Ocular Tissue Separation

#### 3.1.1. Separation of Ocular Substructures

For the separation of contamination-free ocular structures of a small rat eye, it is important to maintain the macroscopic structure of the eye as long as possible. We observed that collecting the aqueous and vitreous humor before enucleation of the eye prevented the eye from collapsing during aspiration. Aspirating the aqueous and vitreous humors lead to clear and consistent sample volumes of ca. 10, and 6 µL, respectively, amenable to HPLC-MS/MS analysis.

In our experience, a dissection of the retina without contamination from melanin containing structures was neither possible from freshly withdrawn nor from frozen rat eyes. Introducing a short fixation step allowed an easy separation of the tissues and led to minimal cross-tissue contamination. An optimal fixation was reached after 90 min in 10% formalin. Shorter fixation times resulted in softer tissues that were hard to separate whereas longer fixation times lead to brittle tissues, especially for the RPE-choroid. Applying this technique, we were able to separate the cornea, iris, ciliary body, lens, retina and the sclera. Iris and ciliary body were combined for compound quantification due to low sample volume. A separation of the cell monolayer RPE from the choroid was mechanically impossible; therefore, these two structures had to be sampled together.

**Figure 1:**
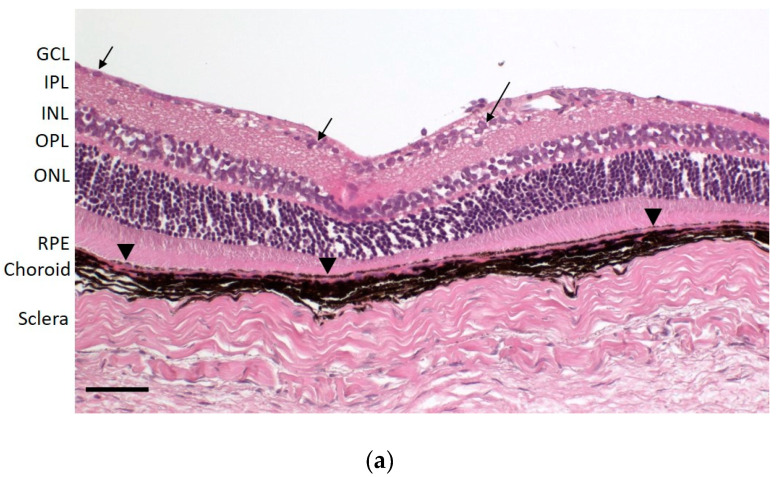

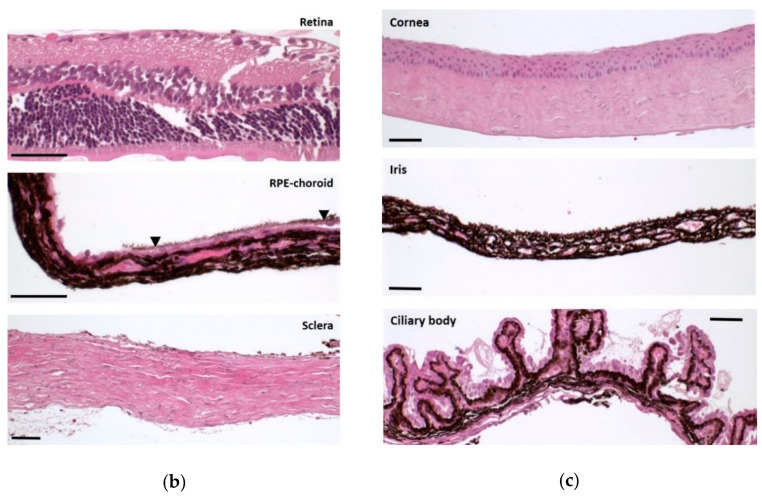
Histology images of BN rat ocular tissues demonstrating the low cross-tissue contamination observed with the presented separation technique. All bars: 50 µm. (**a**) Posterior segment with ganglion cell layer (GCL, black arrows), inner plexiform layer (IPL), inner nuclear layer (INL), outer plexiform layer (ONL), outer nuclear layer (ONL), retinal pigment epithelium (RPE, black arrow heads), choroid, and sclera. (**b**) Separated posterior segment tissue layers from top to bottom: retina, RPE-choroid, sclera. (**c**) Separated anterior segment tissue layers from top to bottom: cornea, iris, ciliary body.

**Figure 2:**
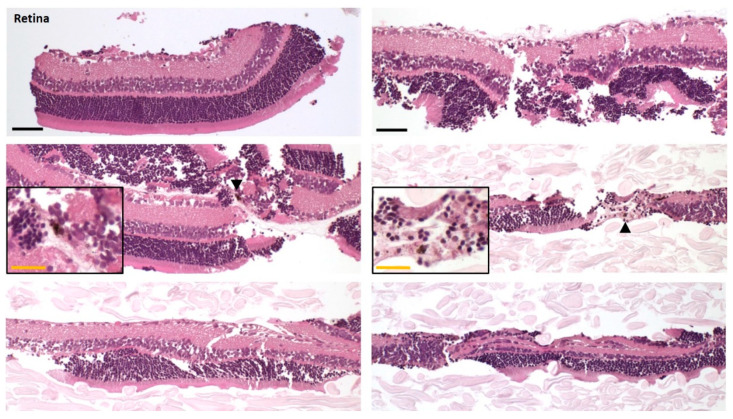
Histology images of retina preparations of several BN rat eyes demonstrating the low cross-tissue contamination observed with the presented separation technique. Black arrowheads show the rarely observed contamination; inserts show these areas magnified. Black bar: 50 µm (applies to all six figures), yellow bar: 25 µm (applies to the insert where shown).

Histological images of the anterior and posterior segment tissues are presented in Figure 1. The contamination observed in different tissue sections was very low and showed low variability; the images shown were chosen to describe the level of contamination representatively. Only the sclera shows some visible contamination; this low level of contamination originates from the choroid and can be detected as pigment deposits on the scleral tissue (Figure 1b). As many targets for posterior segment diseases are located in the retina, we were particularly interested in avoiding contamination of the retina by the underlying, highly pigmented RPE-choroid. Figure 2 shows a series of images of the retina; contaminations were rare and essentially negligible in relation to the size of the isolated pieces of retina. These histological data indicate good reproducibility and high-quality separation.

#### 3.1.2. Melanin Contamination in the Neural Retina

To estimate the contamination of the separated neural retina from the underlying RPE-choroid, we measured the RPE-choroid content in the retinal tissue by measuring absorbance originating from melanin in the RPE-choroid. For this purpose, we prepared a calibration curve with albino retina spiked with pigmented (BN) rat RPE-choroid. The LLOD and LLOQ were 1.2%, and 3.3% of RPE-choroid in the retina (*w*/*w*), respectively.

The melanin content, and thus, the contamination with RPE-choroid of the analyzed pooled retinas was below the LLOD, indicating low contamination. We also attempted to measure the melanin content of the individual retinal samples from which the compound concentration was later determined. Boiling of smaller volumes resulted in high variability in the measurement (data not shown). Therefore, this approach was not suitable to reliably estimate the melanin contamination in the individual retinal samples due to the low sample volume.

### 3.2. Melanin Binding and Drug Extraction from Melanin

We determined the melanin binding of the compounds in vitro with Sepia melanin (1 mg/mL melanin, 1 µM compound). BI 113823 and levofloxacin had clearly higher melanin binding than BI 1026706 and dexamethasone (Table 1). In the same assay, we evaluated the extraction of the compounds from melanin to choose a suitable extraction solvent for the in vivo samples. As the in vivo tissue distribution study was performed by coadministering two compounds at once, the extraction had to be optimized for the respective compound cocktails. The results are listed in Table 1. Extraction was first determined in EtOH:H_2_O (4:1), a solvent commonly used in-house for tissue drug extraction. For the cocktail 1 compounds, dexamethasone extraction was above 50% but levofloxacin extraction was low. Basic conditions (100 mM NH_4_OH in EtOH:H_2_O (4:1)) improved the extraction of levofloxacin. Repeated washing did not improve the extraction of either of these compounds as less than 10% additional extraction was observed with the second and third washings. Therefore, the level of ca. 50% extraction was accepted, and the basic solvent was selected for the in vivo extraction of cocktail 1 compounds. For both compounds in cocktail 2 (BI compounds), the extraction ratio in the original solvent was acceptable and this solvent was used for the in vivo extraction.

### 3.3. Effect of Formalin Fixation on Compound Recovery

We tested the effect of formalin fixation on the recovery of cocktail 1 compounds by performing an additional ocular distribution study in both rat strains and comparing the recovery of compounds from fixed and fresh eyes. In the BN rat, the fixed vs. fresh recovery of each tissue substructure was within the accepted bioanalytical variation of 70–140%, indicating a low effect of formalin fixation on compound recovery (Table 2). Similar variation was observed in the vitreous and aqueous concentrations of left vs. right eye (these were sampled from the fresh eye in all cases). In the WH rat, the recovery was again mostly within the 70–140% range, except for the slightly lower recovery observed in the cornea and posterior segment.

### 3.4. Drug Concentrations in Ocular Tissues

**Figure 3:**
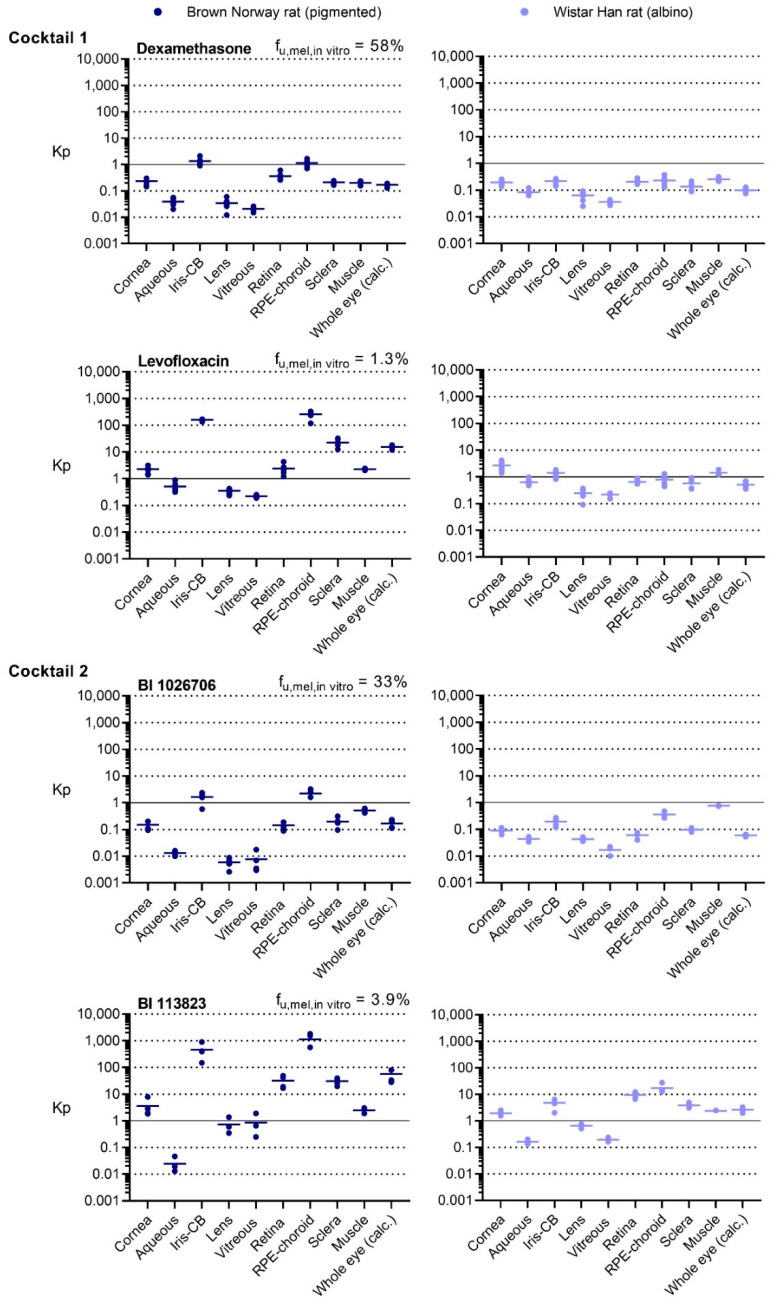
Tissue partitioning (Kp) of the compounds 8 h after oral administration to rats. The individual values for each eye (n = 4–6 eyes) are shown and the mean is presented as a line. Whole eye value was calculated (calc.) with hypothetical volumes (Table S3) for the individual tissues. The measured in vitro melanin binding (1 mg/mL melanin, 1 µM compound) is shown for each compound (f_u,mel,in vitro_).

To investigate the ocular distribution of the drugs, the drug cocktails were administered orally (via gavage) to 2–3 rats/strain (n = 4–6 eyes). Blood and tissues samples were collected to monitor systemic exposure as well as ocular tissue distribution (Figure S1). The tissue results are depicted in Figure 3 as Kp values. Kp values for whole eyes were calculated from the individual tissue values based on hypothetical volumes of the individual tissues. The tissue volumes used as well as the measured tissue concentrations and plasma concentrations can be found in Supplementary Material (Figure S2, Tables S3 and S4).

The inter-individual variability of tissue partitioning for a given compound in a given tissue was low (Figure 3 and Table S4), demonstrating the reproducibility of the method. Varying differences were observed between ocular tissues of the same strain (inter-tissue differences, Figure 3). For all four compounds, the lowest concentrations in both WH and BN rat eyes were detected in the aqueous, vitreous and lens. In WH rats, the highest tissue concentrations were found in the RPE-choroid for the two BI compounds. In contrast, levofloxacin showed the highest concentration in the cornea while dexamethasone had very similar concentrations in all non-humorous tissues. The calculated Kp values largely varied across tissues, the difference between the lowest and highest Kp was 6.3-, 12-, 21- and 100-fold, for dexamethasone, levofloxacin, BI 1026706 and BI 113823, respectively. In BN rats, for all tested compounds, the concentrations in the pigmented tissues iris-CB and RPE-choroid were the highest, leading partially to very extensive lowest-to-highest Kp differences of 65-, 1,100-, 290- and 46,000-fold for dexamethasone, levofloxacin, BI 1026706 and BI 113823, respectively. The 6- to 100-fold Kp differences in albino rats and, more profoundly, the 65- to 46,000-fold differences in pigmented rats illustrate the importance of understanding the exposure in the relevant ocular substructures.

To better understand whether the distribution into non-pigmented ocular structures is comparable between albino and pigmented animals, we calculated Kp ratios between BN and WH rats (BN/WH ratio) (Table 3). For the muscle as a reference tissue, the ratio ranged from 0.68 to 1.6, which is close to the theoretical value of one. As expected, the ratios for iris-CB and RPE-choroid were much higher. There was an apparent link between sclera ratios and the corresponding RPE-choroid ratios, indicating a contamination of this tissue with material from the RPE-choroid which was also visible after tissue separation. Interestingly, the relative concentrations of BI 1026706 were higher in albino than in pigmented animals in the aqueous and vitreous humor and even more pronouncedly in the lens (BN/WH ratios below 1 in Table 3). For BI 113823 we found an increased BN/WH ratio of 4.4 in the vitreous but a low ratio of 0.15 in the aqueous humor. Thus, also non-pigmented ocular tissue partitioning can differ between non-pigmented and pigmented animals.

## 4. Discussion

Although, ocular pharmacokinetic studies in rats can be found in the literature [9,12,13,14,15,23], they rarely report the methods by which the different ocular tissues were isolated and treated after the isolation. In particular, for species with smaller eyes (rat eye diameter ca. 6 mm), the availability of robust, reproducible separation methods is an important cornerstone to gain better understanding of ocular drug distribution and PK/PD. We describe, here, a method allowing a stepwise separation of the anterior and posterior substructures of the rat eye and quantification of small molecules in the separated structures. The reported method, based on a short fixation of the eye in formalin before tissue separation, offers a simple and refined way to isolate the specific tissues with minimal cross-contamination.

Small molecules exhibit high melanin binding potential, stressing the need for clean separation of the posterior segment tissues, retina and RPE-choroid, since a small contamination of the retina with melanin containing tissue could lead to a considerable over-estimation of retinal drug concentrations. Unfortunately, retina and RPE-choroid of the rat eye are very difficult to distinctly separate from one another. We chose to fix the eye in formalin before the separation process, as formalin is known to induce retinal detachment [24] and preserve tissue structure. A short fixation time of 90 min, considerably shorter than the generally used tissue fixation times for histology purposes (overnight or longer), was optimal for our purpose.

Due to the massive concentration differences possible between pigmented and non-pigmented ocular tissues, we applied two methods to investigate retina contaminations with pigmented tissue. While the low melanin content determined in the pooled retinas via the less sensitive photometry was encouraging, the histology of the separated retinas demonstrated the efficiency of the separation process leading to essentially no contaminations with RPE-choroid. We consider this achievement of high importance for an accurate quantification of compounds with melanin binding potential in the retinal tissue.

To understand the effect of fixation on compound recovery, we compared the recovery of the cocktail 1 compounds, levofloxacin and dexamethasone, from fresh and fixed eyes in both rat strains (Table 2). For most of the tissues, fixation had no impact on the recovery, as the concentration in the fixed eye relative to the fresh eye was within the typically accepted error range of analytical methods. For the cornea and the posterior segment in the WH rat, however, we saw a lower recovery of 49 to 66% for both compounds. These lower recoveries could be due to the compound diffusing from these substructures to the formalin or to swelling of the collagen-rich tissues cornea and sclera, as collagenous tissues are known to swell during formalin fixation [25]. It appears likely that the sclera, being the largest tissue of the posterior segment and residing on the surface in direct contact with the formalin, is the major source for the observed lower recovery in the posterior segment. Whether this lower recovery is a result of compound loss or weight gain of the tissues, is out of the interest of this study, considering that the sclera is not a typical target tissue for ocular therapies. As our tissues of interest are mainly in the inner parts of the eye, we consider the method suitable for the reliable analysis of ocular concentrations for our purpose. In case the main tissues of interest for the concentration measurement are in the anterior segment, separating the tissues from fresh eyes may be preferred as this method avoids the possible artifacts (tissue swelling and drug diffusion) with respect to the cornea.

HPLC-MS/MS analysis of the tissue drug concentrations requires the drug to be extracted from the tissue before analysis. Melanin binding of drugs occurs reversibly via different types of electrostatic and hydrophobic interactions between the drug and the melanin polymer depending on the molecular properties of the drug [26,27,28]. It has been observed that some solvents do not extract all of the bound drug from melanin [15,29,30,31], which is explained by the different effects of solvents on the drug-melanin interactions. We evaluated solvent extraction efficiency from melanin in vitro, as direct measurement from in vivo samples would require the use of radiolabeled compounds. Apart from levofloxacin, the compounds showed good extraction in EtOH:H_2_O (4:1). The low recovery of levofloxacin with this solvent and the improvement of the extraction by increasing the basicity of the solvent demonstrated the importance of considering this technical aspect. To improve the accuracy of in vivo concentration measurements, we recommend to optimize compound-specific melanin extraction in vitro.

**Figure 4:**
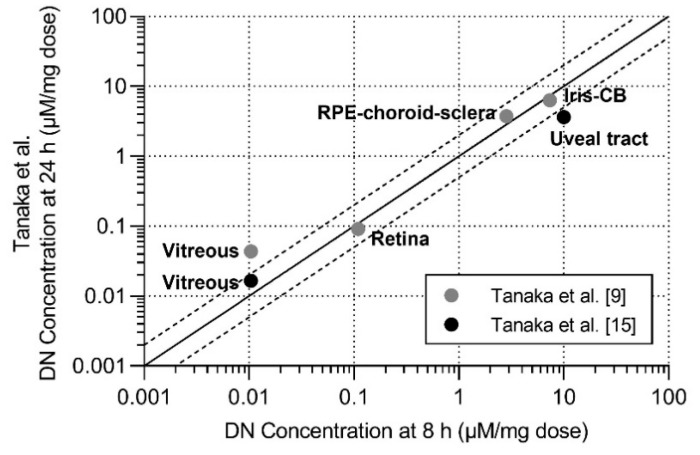
Comparison of dose-normalized (DN) results of levofloxacin ocular distribution between our study (x-axis) and two studies by Tanaka et al. (y-axis) performed with ^14^C-levofloxacin [9,15]. The solid line is the line of identity and the dashed lines represent two-fold deviation.

We applied the tissue separation method to determine the ocular concentrations of levofloxacin and dexamethasone, as well as the two proprietary in-house compounds 8 h after oral administration. We could see substantial ocular distribution with BI 113823 and levofloxacin, as most of the ocular tissues had higher concentrations than the concentration in plasma. The two other compounds, BI 1026706 and dexamethasone, had lower ocular distribution with majority of the tissues having lower concentrations than the concentration in plasma. The lower ocular distribution, especially to the non-pigmented tissues, was also linked with a lower distribution to muscle, a well-perfused tissue described generally as a reference for tissue distribution [17]. The effect of melanin binding on the distribution to the pigmented tissues, RPE-choroid and iris-CB, was extremely evident. The highest differences in Kp between the corresponding BN and WH tissues were observed for the highest in vitro melanin binder levofloxacin in the RPE-choroid (320-fold), whereas the lowest differences (5-fold) were observed for the lowest melanin binder, dexamethasone. The inter-individual variability of the Kp values was low, which demonstrates the reproducibility of the method. Due to the limited concentration data from different tissues of the rat eye available in literature, we cannot thoroughly compare our results to existing data with other tissue isolation methods. However, two ocular distribution studies after oral administration of levofloxacin by Tanaka et al. [9,15] measured the concentrations in ocular substructures of pigmented rats. We compared our concentration results from the studied 8-h time point to their 24-h time point. The tissues combined for the concentration determination differed slightly between their two studies and compared to our investigation; in one of the studies, they measured the concentration from the uveal tract (iris, CB, choroid) and in the other study from the RPE-choroid-sclera. We estimated the concentrations achieved in these tissue combinations, in our study, based on the concentrations from the individual tissues. The good agreement of dose-normalized results between our study and their studies is presented in Figure 4. The differences in concentrations between the corresponding individual tissues are visibly smaller than the differences from one tissue to another. Therefore, considering the different time points used and the different methods employed for the analysis of the concentrations (radioactivity vs. traditional HPLC-MS/MS), the comparison seems very reasonable.

The importance of measuring concentrations directly from the tissue of interest is evident from the observed differences in distribution to individual ocular substructures. Whole-eye exposure, commonly used as a surrogate for ocular tissue distribution, does not correctly describe the exposure in the different substructures (Figure 3). Larger differences among the individual substructures and between the whole-eye and individual tissue concentrations can understandably be seen in pigmented rats, where melanin binding increases the concentrations in the pigmented tissues. Nevertheless, up to 100-fold differences between substructures were also seen in albino animals: For BI 113823, we found a 16-fold lower concentration in the aqueous humor and a 6.5-fold higher concentration in the RPE-choroid compared to the whole eye, demonstrating the difficulty of estimating individual tissue concentrations from the whole-eye concentration. Moreover, extrapolating from one ocular tissue to another, for example from the vitreous to the other tissues of the posterior segment, can be misleading. Retina/vitreous ratios varied from 3.5- to 48-fold in the WH rat and from 11- to 37-fold in the BN rat. The RPE-choroid/vitreous ratios varied from 3.7- to 87-fold and from 55- to 1,300-fold in the WH, and BN rats, respectively.

In addition to the expected and already described concentration differences in RPE-choroid and iris-CB of WH and BN rats, there were also clear inter-strain differences in partitioning to other tissues (Table 3). Kp values of the retina were 1.8- to 3.7-fold higher in the BN rat, whereas differences in vitreous Kp values ranged from 0.45- to 4.4-fold. Aqueous humor Kp values, on the other hand, were always lower in BN rats. Additional investigations with more compounds, including time-dependency studies, are needed to increase the understanding of the drivers and mechanisms of ocular drug distribution. Our data, however, clearly indicate that the distribution into the substructures of the eye is compound specific and exposure differences of albino and pigmented rats are not limited to the pigmented tissues. Therefore, quantification of the compounds directly in the ocular substructures of interest, in the relevant animal strain(s), is needed.

## 5. Conclusions

We have developed a reproducible method for the quantification of drug concentrations in rat ocular tissues by briefly fixing the eye in formalin prior to tissue separation. The method can be applied to both albino and pigmented rats enabling the separation of different ocular tissues with minimal cross-tissue contamination. We investigated possible sources of errors in analyzing concentrations from pigmented eyes and highlighted the importance of analyzing concentrations from individual ocular tissues due to large tissue-specific differences in ocular exposure. Overall, the method facilitates reliable analysis of local drug concentrations in a specific ocular target tissue, an important aspect of estimating PK/PD relationships to support drug discovery and development.

## Figures and Tables

**Table 1:** In vitro melanin binding and extraction of the studied compounds from Sepia melanin (1 mg/mL melanin and 1 µM compound concentration). Individual values presented if two replicates were studied, mean ± standard deviation presented if more than two replicates.

Study/Solvent	Fraction Unbound or Extracted (%)			
	In vivo Cocktail 1		In vivo Cocktail 2	
	Dexamethasone	Levofloxacin	BI 1026706	BI 113823
Melanin binding (unbound fraction (%))	58; 59	1.3 ± 0.5 ^1^	33 ± 5 ^2^	3.9 ± 0.7 ^2^
Extraction EtOH:H_2_O (4:1)	52; 56	5; 18	43; 48	64; 75
Extraction 100 mM NH_4_OH in EtOH:H_2_O (4:1)	51; 53	59 ± 13 ^1^	ns	ns

^1^ Four replicates, ^2^ six replicates, ns = Not studied.

**Table 2:** Recovery of cocktail 1 compounds from substructures of fixed compared to fresh rat eyes. Recovery calculated as percentage of concentration in fixed eye compared to fresh eye. Aqueous and vitreous were sampled from fresh eyes in both cases.

Tissue	Dexamethasone		Levofloxacin	
	BN	WH	BN	WH
Fixed vs. fresh tissue				
Cornea	131 ± 18	66 ± 13	86 ± 20	55 ± 10
Iris-CB	87 ± 7	85 ^1^	138 ± 32	95 ± 21
Lens	91 ± 18	145; 102 ^1^	109 ± 5	161; 110 ^1^
Posterior segment	70 ± 7	49 ± 4	95 ± 16	49 ± 7
Left vs. right eye				
Aqueous	85, 106 ^1^	115 ± 21	108 ± 12	104 ± 10
Vitreous	128 ± 8	122 ± 34	153 ± 35	104 ± 3

^1^ Individual values as data available from only one or two samples.

**Table 3:** Ratio of Kp values between pigmented BN and albino WH rat (BN/WH ratio). The ratios were calculated from the average Kp values. Whole eye value was calculated (calc.) with hypothetical volumes (Table S3) for the individual substructures.

Tissue	Dexamethasone	Levofloxacin	BI 1026706	BI 113823
Cornea	1.2	0.85	1.7	1.9
Aqueous	0.47	0.80	0.31	0.15
Iris-CB	6.2	113	8.5	96
Lens	0.55	1.4	0.14	1.1
Vitreous	0.57	1.0	0.45	4.4
Retina	1.8	3.7	2.4	3.4
RPE-choroid	5.0	320	6.2	66
Sclera	1.6	40	2.1	8.0
Muscle	0.8	1.6	0.68	1.0
Whole eye (calc.)	1.7	30	2.8	21

## Supplementary Materials

The following are available online at https://www.mdpi.com/1999-4923/12/12/1174/s1, Figure S1: Step-by-step rat ocular tissue separation., Table S1: Tissue-dependent extraction ratios used for tissue sample preparation for HPLC-MS/MS analysis., Table S2: Calibration ranges (nmol/L) of HPLC-MS/MS analysis of the in vivo plasma and tissue concentration., Figure S2: Plasma concentration-time profiles of the individual Brown Norway (BN) and Wistar Han (WH) rats after oral delivery (p.o.)., Table S3: Tissue volumes used for the calculation of whole eye/plasma concentration ratios., Table S4: Average concentrations measured from ocular tissues and muscle.
