# Supplementary material for: Quantification of Drugs in Distinctly Separated Ocular Substructures of Albino and Pigmented Rats
Source: Pharmaceutics. 2020 Dec 2;12(12):1174. doi: 10.3390/pharmaceutics12121174 (PMC7760391; doi:10.3390/pharmaceutics12121174)
Supplement: Supplementary file 1 [file pharmaceutics-12-01174-s001.pdf]

pharmaceutics

Supplementary Material: Quantification of Drugs in Distinctly Separated Ocular Substructures of Albino and Pigmented Rats

Anna-Kaisa Rimpelä, Michel Garneau, Katja S. Baum-Kroker, Tanja Schönberger, Frank Runge and Achim Sauer

Separation of ocular tissues

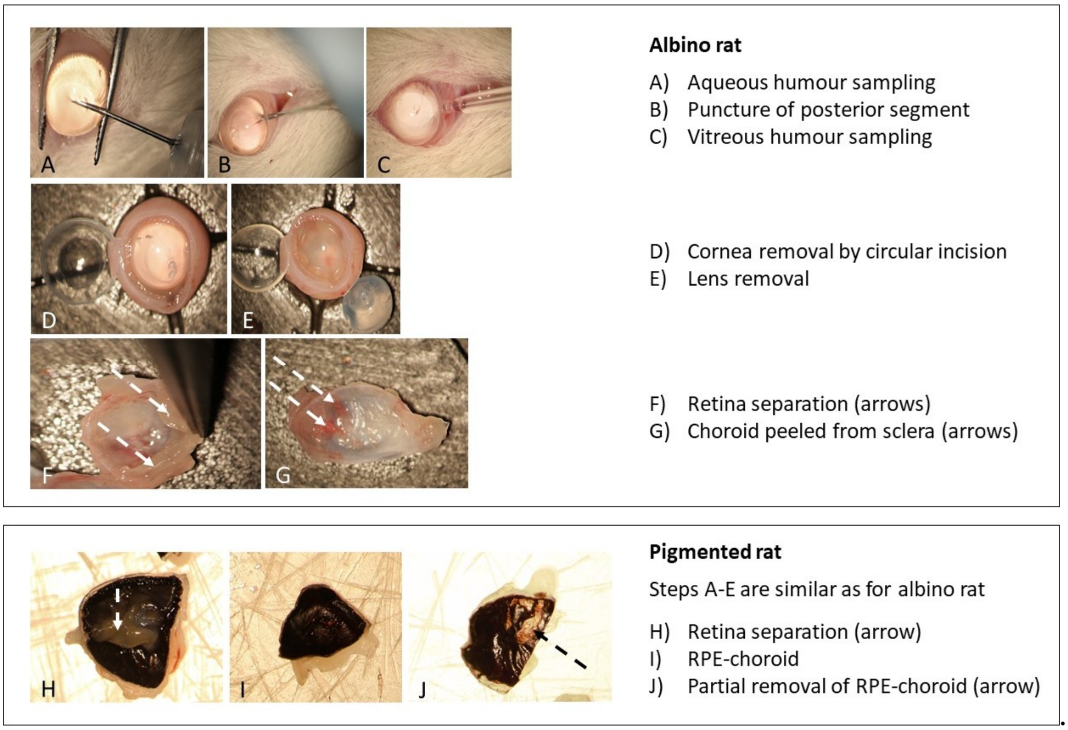

Figure S1. Step-by-step rat ocular tissue separation. Magnification 6-10x.

HPLC-MS/MS for in vivo samples

Table S1. Tissue-dependent extraction ratios used for tissue sample preparation for HPLC-MS/MS analysis.

Tissue	Extraction ratio (µL/mg tissue)
Cornea	100
Aqueous	10
Iris-CB	100 or 200
Lens	10
Vitreous	10, 20 or 40
Retina	40 or 50
RPE-choroid	250
Sclera	100

Table S2. Calibration ranges (nmol/L) of HPLC-MS/MS analysis of the *in vivo* plasma and tissue concentration.

	WH rat		BN rat	
	**Dexamethasone**	**Levofloxacin**	**Dexamethasone**	**Levofloxacin**
Plasma	1-5000	1-5000	1-5000	2-5000
Tissues	0.05-500	0.05-500	0.1-500	0.2-500
	**BI 113823**	**BI 1026706**	**BI 113823**	**BI 1026706**
Plasma	0.5-5000	1-5000	0.5-5000	0.5-5000
Tissues	0.05-1000	0.1-1000	0.1-1000	0.05-1000

Plasma concentrations

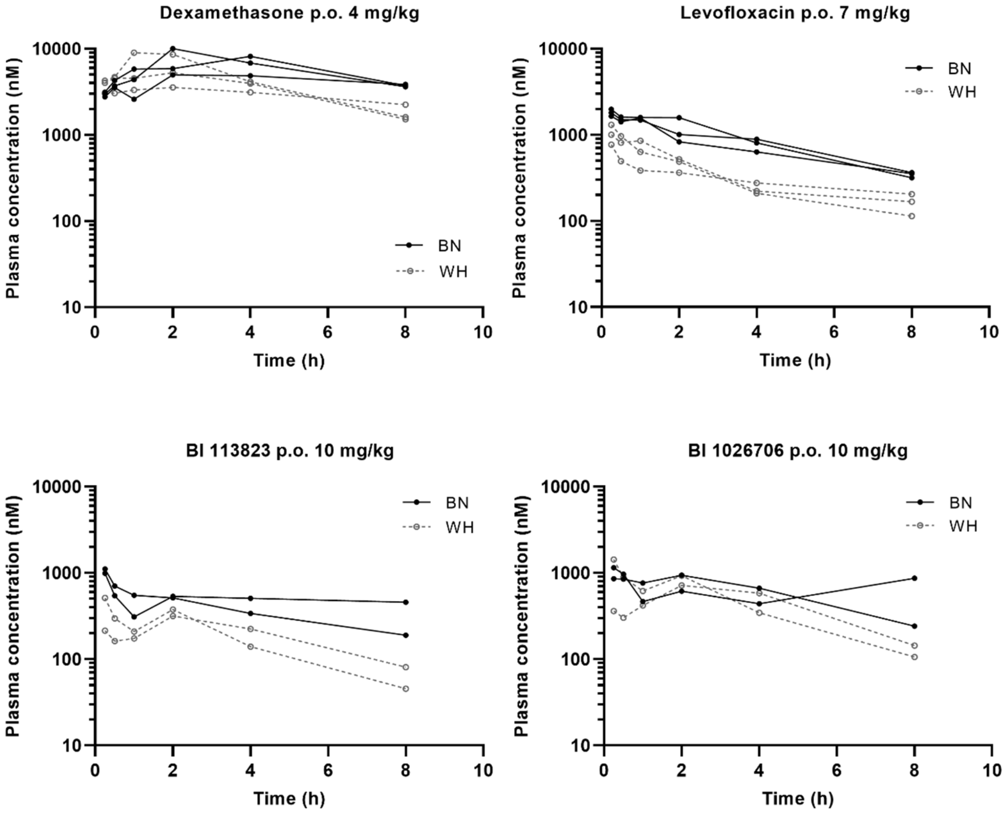

Figure S2. Plasma concentration-time profiles of the individual Brown Norway (BN) and Wistar Han (WH) rats after oral delivery (p.o.). Dexamethasone and levofloxacin: n=3 rats/strain, BI 113823 and BI 1026706: n=2 rats/strain.

Estimated tissue volumes and calculation of whole-eye tissue/plasma ratio

We estimated whole eye/plasma concentration ratios (Kp) by calculating the whole eye concentration from the individual tissue concentrations based on hypothetical tissue volumes. As the rat eye tissue volumes are known to change with age, the hypothetical values corresponding with in-house weight measurements and literature values available were used. The volumes used are presented in Table S3.

Table S3. Tissue volumes used for the calculation of whole eye/plasma concentration ratios.

Tissue	Volume (μL)
Cornea	5
Aqueous	15
Iris-CB	3
Lens	30
Vitreous	35
Retina	15
RPE-choroid	4
Sclera	15
Total	122

Original tissue concentrations

Table S4. Average concentrations (nM) measured from ocular tissues and muscle.

Dexamethasone	BN rat (n=3 rats, 6 eyes)			WH rat (n=3 rats, 6 eyes)		
Tissue	Mean	SD	%CV	Mean	SD	%CV
Cornea	867	232	27	339	78	23
Aqueous	143	43	30	151	58	39
Iris-CB	5060	1410	28	379	72	19
Lens	129	54	42	106	30	28
Vitreous	76.9	13	16	64.1	4.2	7
Retina	1320	418	32	356	56	16
RPE-choroid	4250	1170	28	406	139	34
Sclera	781	126	16	238	78	33
Muscle	753	113	15	465	164	35
**Levofloxacin**	**BN rat (n=3 rats, 6 eyes)**			**WH rat (n=3 rats, 6 eyes)**		
**Tissue**	**Mean**	**SD**	**%CV**	**Mean**	**SD**	**%CV**
Cornea	783	230	29	415	143	34
Aqueous	171	57	33	104	46	45
Iris-CB	54500	6260	11	214	38	18
Lens	121	27	22	37.9	14	38
Vitreous	76.9	7.5	10	35.1	12	34
Retina	818	282	34	102	13	13
RPE-choroid	88000	25300	29	120	35	29
Sclera	7690	1920	25	87.9	32	36
Muscle	776	45	6	243	100	41
**BI 1026706**	**BN rat (n=2 rats, 4 eyes)**			**WH rat (n=2 rats, 4 eyes)**		
**Tissue**	**Mean**	**SD**	**%CV**	**Mean**	**SD**	**%CV**
Cornea	67.6	21	31	11.3	3.4	30
Aqueous	6.67	3.0	45	5.38	1.2	23
Iris-CB	733	369	50	22.9	4.3	19
Lens	2.62	1.1	42	5.23	0.36	7
Vitreous	3.44	1.9	56	2.34	0.91	39
Retina	68.4	26	38	7.28	1.2	16
RPE-choroid	1030	347	34	43.8	6.8	15
Sclera	90.4	38	42	11.8	2.3	19
Muscle	365; 147 *			78.5; 111 *		
**BI 113823**	**BN rat (n=2 rats, 4 eyes)**			**WH rat (n=2 rats, 4 eyes)**		
**Tissue**	**Mean**	**SD**	**%CV**	**Mean**	**SD**	**%CV**
Cornea	940	345	37	122	35	28
Aqueous	6.82	2.2	33	10.1	2.4	24

Iris-CB	125000	53600	43	278	73	26
Lens	202	66	33	41	9.8	24
Vitreous	224	107	48	13	4.7	37
Retina	8440	596	7	567	74	13
RPE-choroid	29100	35000	12	1010	211	21
Sclera	8940	2000	22	233	34	14
Muscle	860; 575 *			112; 187 *		

* Individual values of muscle concentrations of BI 113823 and BI 1026706 are reported (n=2 rats)
